# The direct effect of SARS-CoV-2 virus vaccination on human ovarian granulosa cells explains menstrual irregularities

**DOI:** 10.1038/s41541-024-00911-2

**Published:** 2024-06-26

**Authors:** Hadas Bar-Joseph, Yael Raz, Anat Eldar-Boock, Nadav Michaan, Yoel Angel, Esther Saiag, Luba Nemerovsky, Ido Ben-Ami, Ruth Shalgi, Dan Grisaru

**Affiliations:** 1 TMCR Unit, The Veterinary Service Center, Faculty of Medicine, Tel Aviv University, Tel Aviv, 69978 Israel; 2 Department of Gynecologic Oncology, Lis Maternity Hospital, Tel Aviv Sourasky Medical Center, Tel Aviv 6423906, Israel. Affiliated to the Faculty of Medical & Health Sciences, Tel Aviv University, Tel Aviv, Israel; 3 Tel Aviv Sourasky Medical Center, Tel Aviv, Israel, The Faculty of Medical & Health Sciences and the Coller School of Management, Tel Aviv University, Tel Aviv, Israel; 4 Tel Aviv Medical Center, Tel Aviv, Israel; 5 Department of Cell and Developmental Biology, The Faculty of Medical & Health Sciences, Tel Aviv University, Tel-Aviv, Israel; 6 Department of Obstetrics and Gynecology, IVF and Infertility Unit, Sha’are Zedek Medical Center, The Hebrew University Medical School of Jerusalem, Jerusalem, 9103102 Israel

**Keywords:** RNA vaccines, Translational research

## Abstract

Following administration of the SARS-CoV-2 vaccine, many women worldwide reported short-term menstrual irregularities. Although menstrual bleeding, “the fifth vital sign”, is experienced by more than 300 million people on any given day worldwide, these changes were only partially studied. Irregular periods are important well beyond fertility and the discomfort they impose; they are associated with the risk of cardiovascular morbidity, chronic diseases, and premature mortality. Pre-clinical examination of the vaccine polymeric envelope indicates its accumulation in the ovaries. The somatic endocrine cells of the ovarian follicle - the granulosa cells (GCs)—participate in the strict hypothalamic-pituitary-ovarian (HPO) feedback loop that governs the menstrual cycle via endocrine and paracrine regulators, as AMH and Inhibins. We aimed to unravel the direct effect of the COVID-19 vaccine on GCs and link their post-vaccine activity to changes in menstrual patterns. Human primary GCs exposed in-vitro to the Pfizer COVID-19 vaccine BNT162b2, demonstrated no change in their viability but altered mRNA transcripts, specifically of the regulatory key factors: InhibinB was upregulated, whereas AMH was downregulated. We further examined pre- and post-vaccination blood samples from individual women and found a 2–3 folds change in the post-vaccination FSH/InhibinB protein level ratio, compared to their pre-vaccination values. This altered expression of InhibinB could significantly impact the HPO axis in vaccinated women and may ultimately influence the endometrium cyclicity, manifested clinically by the commonly reported changes in menstrual bleeding patterns.

## Introduction

Historically, research concerning menstrual disorders has been sparse, largely due to unscientific notions linking menstrual bleeding with pollution and impurity. The COVID-19 pandemic revealed a critical gap in female health science, aggravating anxiety, ignorance, and vaccine hesitancy. During the COVID-19 pandemic, millions of women worldwide were vaccinated against the SARS-CoV-2 virus. Though pre-clinical and retrospective clinical data agree as to the vaccine’s safety for fertility and pregnancy^1,2^, not long after administration of the first and second doses many women reported changes in their menstrual bleeding patterns. These reports were followed by peer-reviewed publications supporting these phenomena^3–7^, regardless of the vaccine brand^8–11^. The evidence points to short-term but statistically significant temporary changes in cycle length and flow^2,4,8,9^, most of which resume within one cycle^1,10–12^. These changes could be associated with multiple characteristics such as women’s age, contraception type in use, ethnicity, the severity of other vaccine side effects, and more^8,12^. Nonetheless, the link between the day of the cycle at vaccination and menstrual changes is controversial^4,8^.

A link between menstrual irregularities and vaccine administration was demonstrated as early as 1913, tracking the typhoid vaccine^2^; since then, it has been shown that hepatitis B and papilloma viruses vaccines affect the menstrual cycle as well^2,4,8,13,14^. It was suggested that this side effect is a result of the immune response to the vaccine, activation of endometrial immune cells that regulate the generation of the endometrium, or immunological influence on the secretion of follicular stimulating hormone (FSH) and luteinizing hormone (LH)^4,9,15^. Examination of the COVID-19 vaccine pointed to an immunologic response, manifested by cytokine release (as Interleukin-8; IL-8)^16^. However, a direct effect of the vaccine on the ovary should be examined, as recent papers showed that ovarian cells could be directly infected by the SARS-CoV-2 virus^17^, and that the vaccine caused a biological response similar to that of prolactin signaling, which may lead to short-term menstrual irregularities^18^.

Pfizer-BioNTech’s COVID-19 vaccine, BNT162b2 (commercially named COMIRNATY®; “vaccine”), was the first to be authorized by the FDA for wide use. The vaccine uses a clinically validated lipid nanoparticle (LNP) technology, developed by Acuitas therapeutics Inc^19^. In its “Final Report” to the FDA^20^, Acuitas followed the distribution and accumulation of an LNP envelope containing tagged-mRNA (LNP vehicle) in the body of research-model rats and suggested the ovaries as one of the four organs that accumulated the LNP vehicle.

The ovary’s main functional unit is the follicle. It is comprised of an oocyte surrounded by somatic endocrine granulosa cells (GCs) that support the oocyte during folliculogenesis^21,22^. GCs are part of the hypothalamic-pituitary-ovarian (HPO) axis^23–25^ that governs the reproductive system activity^26^. The menstrual cycle can be easily disrupted, for example due to stress and poor nutrition^9,15^.

We hypothesized that the menstrual changes in post-vaccinated women may result from a direct effect of the vaccine on the GCs, leading to modifications in the expression and secretion of follicular hormonal regulators and thus affecting the cycle. InhibinB is produced primarily by GCs of FSH-dependent growing antral follicles and secreted predominantly during the follicular phase of the cycle (before ovulation)^27^. There is little evidence of a correlation between serum levels of InhibinB and menstrual cycle parameters such as flow and bleeding duration. However, as InhibinB is a pivotal participant in the HPO feedback loop^24,25^, that is correlated with cycle length^28^, we focused on it.

## Results

We aimed to determine the direct effect of BNT162b2 COVID-19 vaccine on ovarian GCs and suggest it contributes to post-vaccination menstrual changes.

We exposed in-vitro human primary GCs (hpGCs), obtained from women undergoing IVF treatments, to two concentrations of the vaccine (“injected dose” or “end-organ dose”; see “Methods”) for 24 or 48 h. To overcome biases resulting from differences in women’ characteristics and IVF protocols (detailed in Supplementary Table 1), we pooled hpGCs from more than one woman in each of our experiments.

**Fig. 1:**
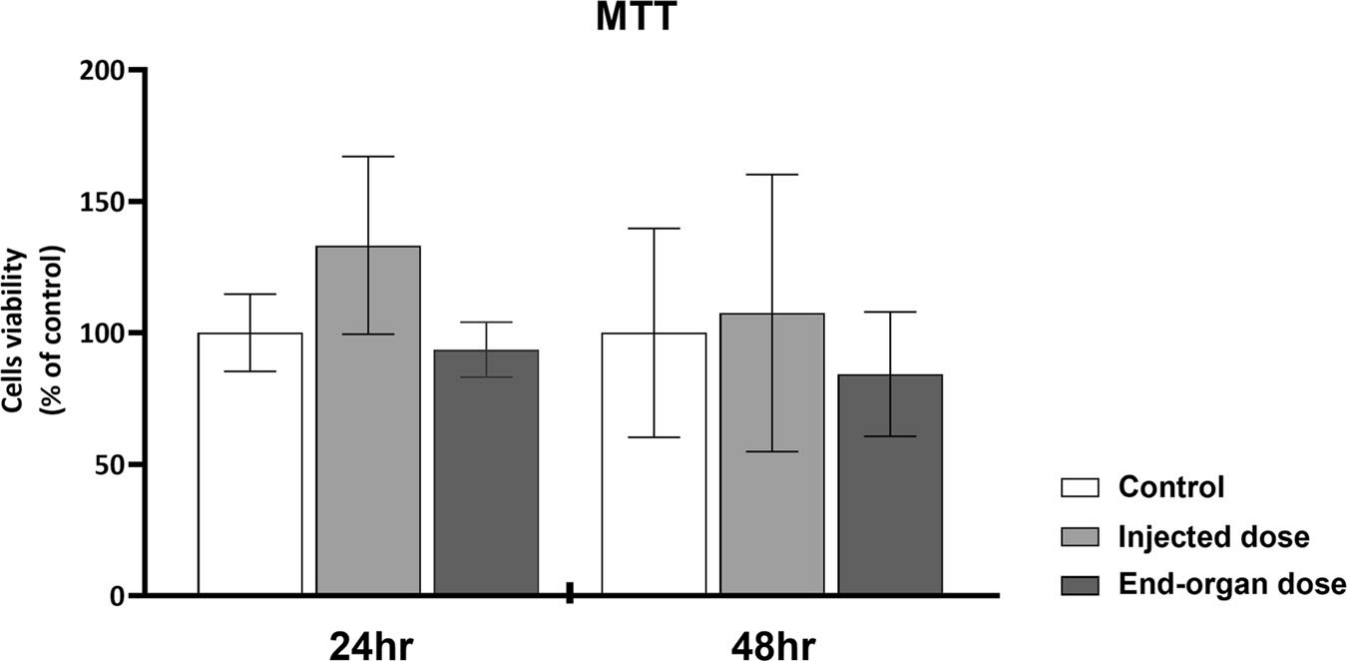
Human primary GCs (hpGCs) vitality after exposure to BNT162b2 COVID-19 vaccine. hpGCs (cells were cultured in pools) were exposed to 2 vaccine concentrations: “injected dose” and “end-organ dose”. Non-treated cells served as control. hpGCs were harvested for vitality analysis using MTT assay at 24 or 48 h. Data are presented as Mean ± SEM of relative expression. *P < 0.05—significantly different from control value. Data were analyzed by Kruskal–Wallis followed by FDR correction for multiple comparisons. Each experiment was conducted three times in duplicates.

First, we examined whether the vaccine inflicts a toxic effect on the vitality of the cells. Using an MTT test (Fig. 1), we showed that the cells’ vitality is not compromised by the vaccine, regardless of dose concentration or time of exposure.

**Fig. 2:**
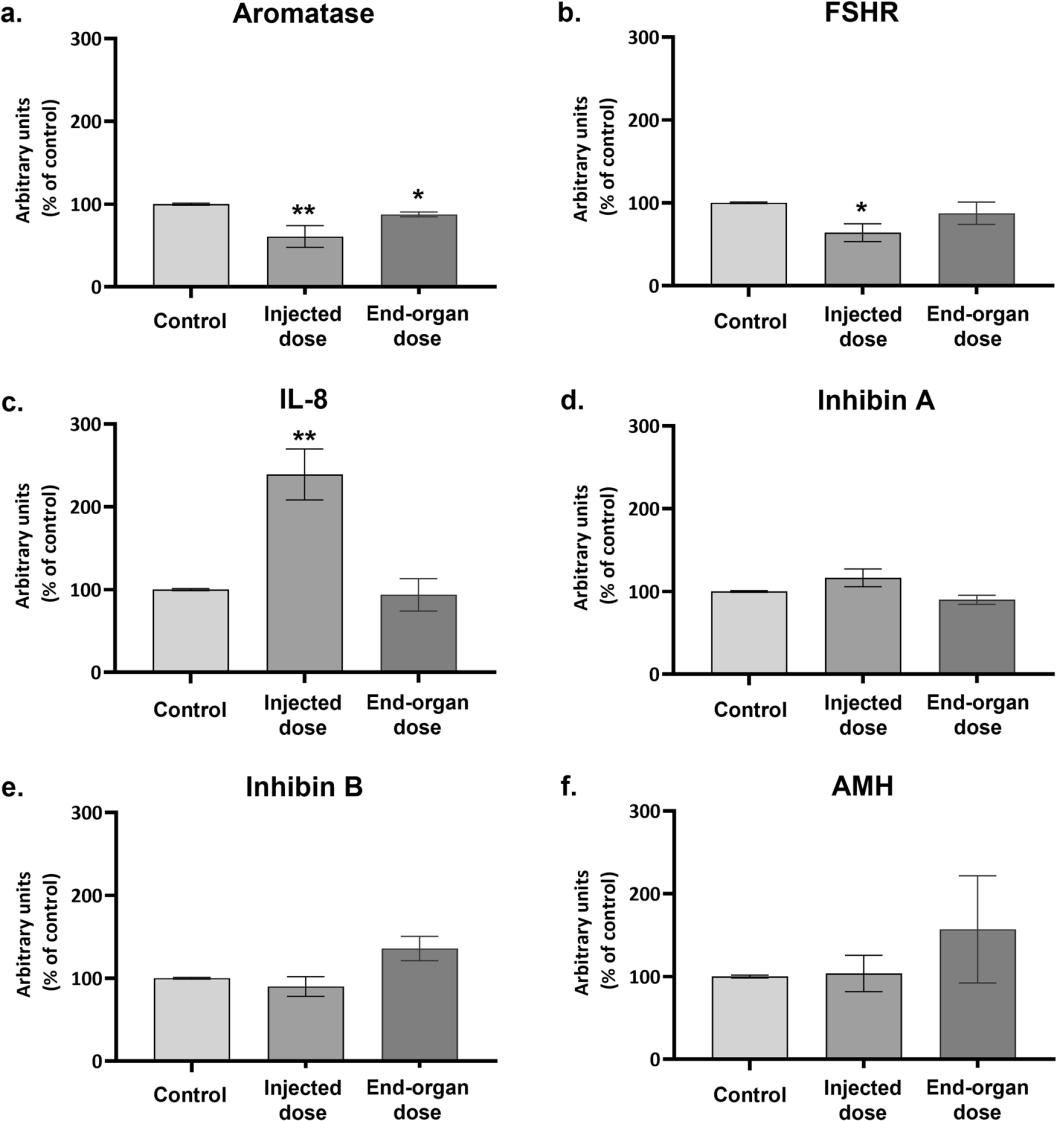
mRNA Expression of selected genes in hpGCs Following 24-hour Exposure to BNT162b2 COVID-19 Vaccine. **a**–**f** hpGCs (cells were cultured in pools) were exposed to 2 vaccine concentrations: “injected dose” and “end-organ dose” for 24 h and harvested for mRNA analysis via qPCR. Non-treated cells served as control. Data are presented as Mean ± SEM of relative expression. *PV < 0.05—significantly different from control value. Data were analyzed by Kruskal–Wallis followed by FDR correction for multiple comparisons. Experiments were conducted at least five times.

Next, we followed the level of mRNA transcripts of genes associated with GCs activity. While 24 h of exposure to the injected dose of the vaccine (Fig. 2) caused a decrease in the mRNA levels of *aromatase* and *FSHR* and a prominent increase in *IL-8* mRNA level (no change was detected in *InhibinA, InhibinB* or *AMH* mRNA levels), exposure to the end-organ dose led to an increase of *InhibinB* mRNA level that did not reach a statistical significance (PV = 0.061), concomitant with downregulation of aromatase level.

**Fig. 3:**
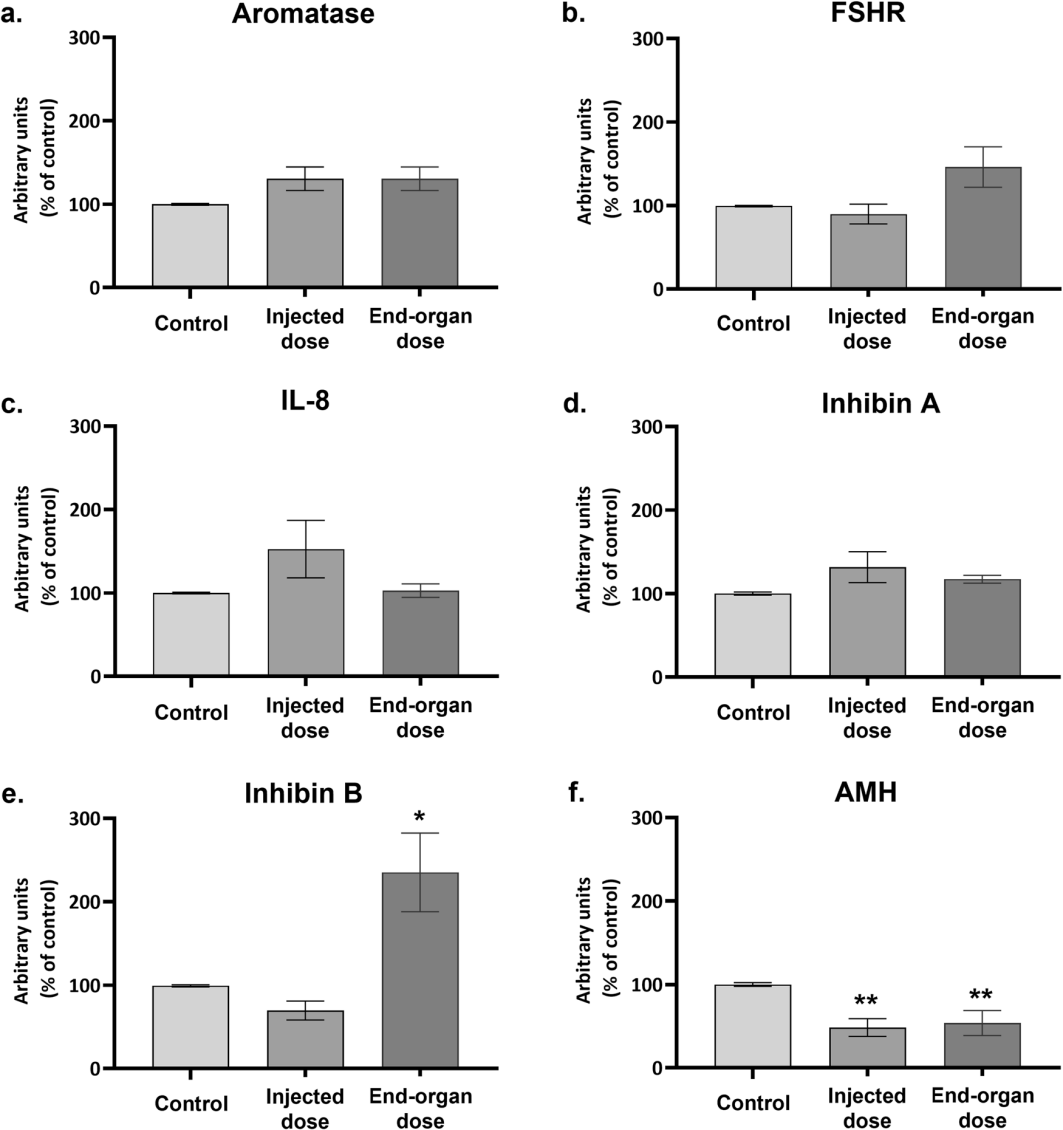
mRNA Expression of selected genes in hpGCs Following 48-hour Exposure to BNT162b2 COVID-19 Vaccine. **a**–**f** hpGCs (cells were cultured in pools) were exposed to 2 vaccine concentrations: “injected dose” and “end-organ dose”. Non-treated cells served as control. Forty-eight hours later, hpGCs were harvested for mRNA analysis. The corresponding mRNAs were subjected to qPCR analysis with specific primers for aromatase, FSHR, IL-8, AMH, InhibinA, and InhibinB and calibrated by HPRT1. Data are presented as Mean ± SEM of relative expression. *PV < 0.05—significantly different from control value. Data were analyzed by Kruskal–Wallis followed by FDR correction for multiple comparisons. Experiments were conducted at least five times.

After 48 h of exposure (Fig. 3), the injected dose no longer influenced the mRNA levels of *aromatase, FSHR, IL-8*, or *InhibinA*. These mRNA transcripts were not affected by the end-organ dose as well. However, the end-organ dose caused a profound increase (more than 200%) in *InhibinB* mRNA level. *AMH* level was downregulated at both doses.

Next, we measured the level of InhibinB protein secreted from the hpGCs into the culture medium, in response to vaccine exposure, and detected an increase in its level (112, 121, and 134% in three independent experiments, compared to control) after 48 h of exposure to the end-organ dose, matching the increase in its mRNA level. There was no detectible change in InhibinA protein level in the culture medium in either dose or exposure duration to the vaccine (data not shown).

**Fig. 4:**
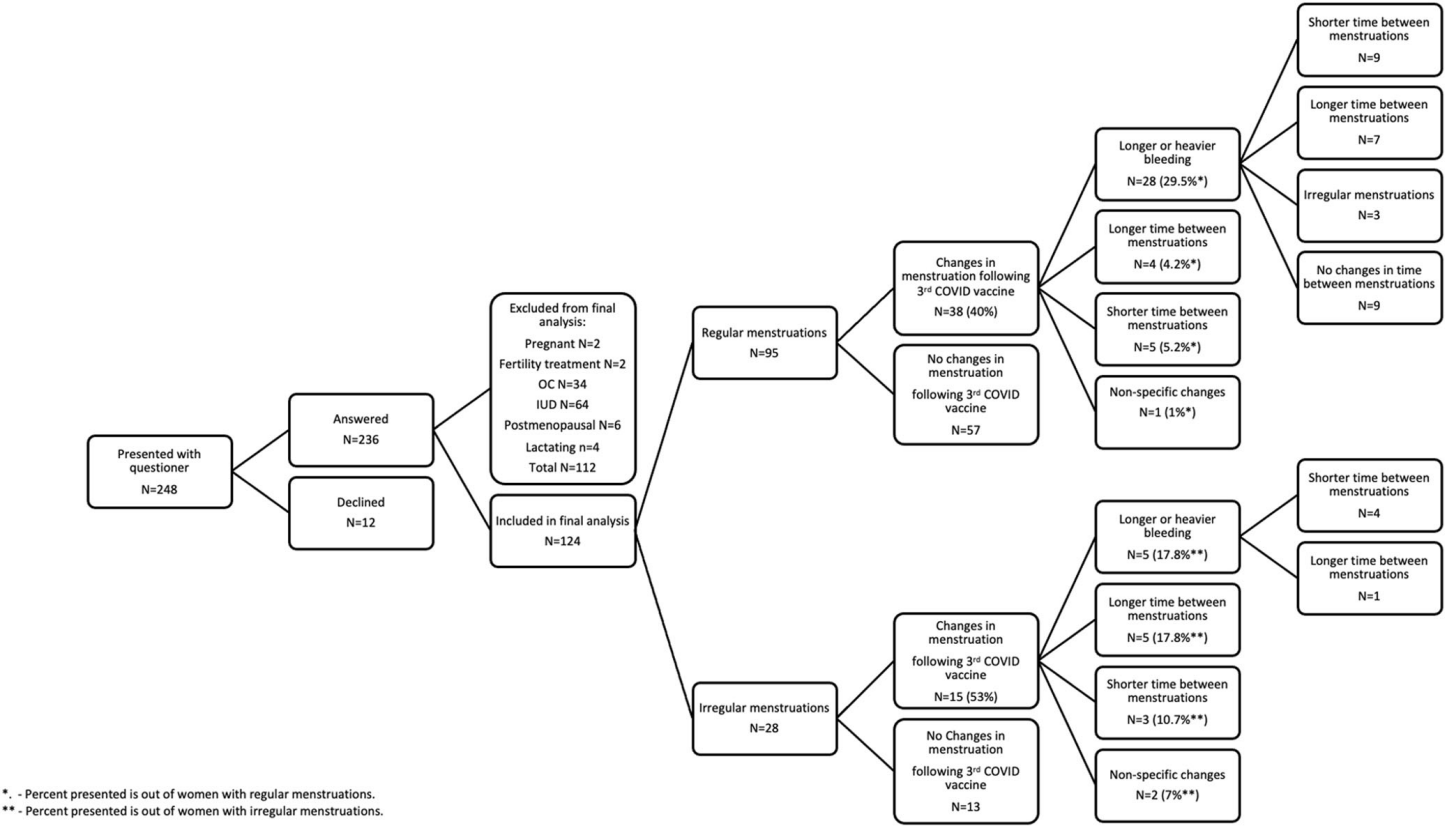
Menstrual changes post 3rd dose of the COVID-19 vaccination questionnaire summary. Two hundred forty-eight women, attending Sourasky Medical Center were presented with an electronic questionnaire ~4 months after vaccination, following changes in their menstrual cycle after COVID-19 vaccine administration. Two hundred thirty-six completed the survey, 124 of them were included. OC oral contraceptives, IUD intrauterine device.

To accompany our in-vitro experimental investigation, we interviewed women attending the Sourasky Medical Center ~4 months after receiving their third dose of the BNT162b2 COVID-19 vaccine. Out of 236 women who answered our electronic questionnaire (Fig. 4), 112 were excluded due to various reasons as pregnancy or menopause, and 124 were included in our final analysis. Forty percent of women with regular menstruations and 53% of women with irregular menstruations reported various changes in their cycle length and bleeding patterns following administration of the 3rd vaccine dose.

**Fig. 5:**
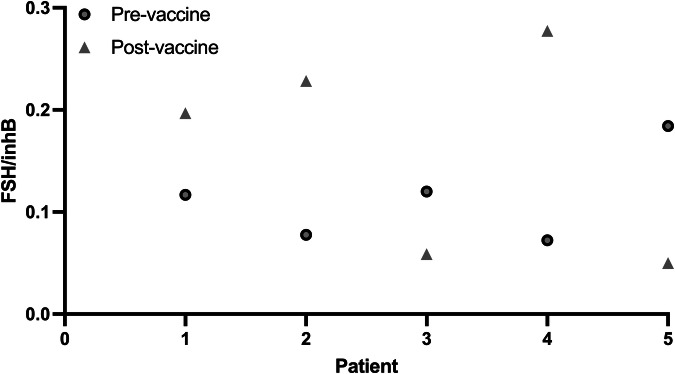
FSH/InhibinB ratio changes in response to administration of the COVID-19 vaccine. FSH/InhibinB ratio in 5 women vaccinated with the 3rd dose of the COVID-19 vaccine and who reported subsequent changes in menstrual bleeding patterns.

Finally, we asked whether the detected increase in inhibinB, following a direct vaccine exposure, can also be tracked in vaccinated women and point to its role in menstrual irregularities. To that end, we analyzed blood samples from five women before and ~1 month after 3rd dose vaccination. All women reported changes in their menstrual bleeding pattern post the vaccine. Blood was collected without recording the day of the menstrual cycle. Since FSH/InhibinB present a relatively stable ratio, independent of the day of cycle^29^, and as each woman has her own FSH/InhibinB ratio that is steady, not only throughout the cycle but in following cycles^30,31^, we followed that ratio. We found that for all women tested, the FSH/InhibinB ratio was changed 2–3 folds post vaccination (Fig. 5).

## Discussion

During the COVID-19 pandemic, millions of women worldwide were vaccinated against the SARS-CoV-2 virus. Not long after, women administered with the first and second doses reported changes in their menstrual cycle^1,4,5^. The influence of vaccines on women’s menstrual bleedings was previously established, as in the vaccines against Hepatitis B and Papilloma viruses^2,9,13,14^ where activation of the immune system, inflammation, and disturbed regulation of the uterus regeneration were suggested as the cause^4,9,15^.

Studies that followed ovarian function in vaccinated women undergoing IVF treatments indicate that the vaccine doesn’t affect ovarian function or oocyte quality (oocyte maturation or fertilization rates following mRNA-based vaccines)^32^, even when anti-COVID-19 IgG antibodies were detected in the follicular fluids (FF)^33^. Moreover, ovarian reserve post vaccination, assessed by serum AMH levels^34,35^ as well as antral follicle count and hormonal serum leves^36^, were not changed. None the less, irregular periods are important well beyond fertility; they cause discomfort and emotional stress, which are often being ignored. In addition, they are associated with the risk of cardiovascular morbidity, chronic diseases, and premature mortality. The current study shows that changes in the menstrual cycle occur after the 3rd dose (~6 months after the second dose) and not only after the first and second vaccinations, as in most of the reports^1,10,11,12^.

Regulated by the HPO axis^23^, the menstrual cycle is comprised of three phases: follicular, ovulatory, and luteal. The follicular phase - from the beginning of the menstrual bleeding to ovulation - governed by FSH promoting follicular growth, and the luteal phase - from ovulation to the next menstrual bleeding - regulated by a surge of LH promoting corpus luteum formation. The normal length of ovulatory cycles (between 21 and 35 days), is usually derived from varying lengths of the follicular phase^26^. In contrast, the luteal phase is relatively constant in its duration (~14 days)^26^.

GCs, the somatic endocrine cells of the follicle, participate in the strict HPO feedback loop^24,25^ in the following manner: FSH stimulates GCs via FSH receptor (FSHR) to secrete endocrine and paracrine regulators, as estrogen (produced by Aromatase within the GCs), AMH and Inhibins. In turn, these hormones regulate FSH, by either directly reducing its synthesis and secretion from the hypophysis (estrogen and inhibins^24,25^) or indirectly reducing follicle’s sensitivity to FSH (AMH^37,38^). As GCs play a role in the regulation of the HPO axis, and following the Acutias “Final report”, indicating the LNP vehicle accumulates in ovaries, we aimed to explore if the vaccine can affect directly the expression of ovarian regulators, that in turn may explain post-vaccination changes in menstrual cycles.

To assess the direct effect of the vaccine on the ovary, we pooled hpGCs isolated from several women, thereby mitigating biases due to individual physiological differences and treatment protocols. We examined two vaccine concentrations to determine their effects: (i) 0.5 μg/ml (‘injected dose’) to evaluate cell toxicity and (ii) 0.5 pg/ml (‘end-organ dose’), representing less than what is estimated to accumulate in the human ovary.

We first aimed to find whether the vaccine is toxic to the cells, using a 1000-times higher dose than in the end-organ. We found that the hpGCs’ viability was not compromised after 24 or 48 h of exposure to either the injected or the end-organ doses. Thus, we concluded that the findings gathered in this work were not a result of impairment to the cells vitality but rather related to changes in their activity.

To support our hypothesis, we examined the mRNA level of GCs’ activity-related genes. A 24-h stimulation with the vaccine injected dose caused a decrease in the mRNA levels of *aromatase* and *FSHR*, reflecting their reduced activity and receptivity to FSH. Together with the robust increase in the mRNA level of the pro-inflammatory chemokine *IL-8*, which acts to resolve the inflammatory stimulus and promote healing^39^, these outcomes most likely represent an acute stress phase experienced by the cells. Such cellular response was expected following a direct exposure to the injected dose of the vaccine. Interestingly, the end-organ dose did not cause a notable change in *aromatase, FSHR* or *IL-8* expression but led to an increase in *InhibinB* mRNA level that did not reach a statistical significance and was not yet reflected in the secreted protein level.

We continued to follow the hpGCs after a 48-h exposure to the vaccine, as was measured in the “Final report”. At this time point, the stress-related changes detected after the 24-h exposure to the injected dose reverted to their baseline state, pointing to a recovery of the cells’ activity and responsiveness. However, exposure to the end-organ dose resulted in a prominent elevation in *InhibinB* mRNA level. The level of the secreted protein displayed a similar trend, further supporting our results. Even though secretion of InhibinB into the culture medium was elevated compared to control in 3 independent experiments, it was not statistically significant. This may be due to the differences in InhibinB concentration (as measured by ELISA) of the 3 independent controls, caused by different attachment and survival rates of the hpGCs during the culture period. These differences are reflected in the high STDEV of the control group, which in turn affected the *P* value. Additionally, we might have detected a more prominent elevation in the secreted protein had we allowed a longer culture period, as proteins are expressed after mRNAs. Nonetheless, these results support the observed elevated-mRNA levels and imply a rise of InhibinB levels in post-vaccinated women’s sera. The latter may cause a disruption of the hormonal axis rooted at the base of the reported changes in menstrual cycle, implying an altered InhibinB level ~1 month after vaccination.

We further followed other participants in the HPO axis.

InhibinA, another member of the Inhibin family, is involved in regulating FSH and LH secretion. As it is expressed mainly by the corpus luteum^22^ we were not surprised to find no effect of the vaccine on its expression in hpGCs.

AMH, produced exclusively by GCs, mainly of small growing follicles up to small antral follicles^37,38,40^, contributes to the menstrual cycle by affecting other follicles in a paracrine manner. It downregulates the FSHR level in pre-antral follicles, and inhibit the activation of primordial follicles from the ovarian pool^29^. Even though we used hpGCs that were isolated from preovulatory follicles, we did detect *AMH* expression. This might be due to the fact that in IVF, oocytes are isolated from small antral follicles as well, or due to a low basal expression of *AMH* by large antral follicles^41^. We observed a significant reduction in *AMH* transcripts in both vaccine doses, 48 h after administration in-vitro. It implies to a similar reduction in AMH expression in smaller follicles in the ovary following vaccination, that may result in a larger population of hormonally-active follicles, that can cause an even higher serum level of InhibinB and disruption to the cycle. However, as showed, it does not represent a clinically diminished primordial reservoir^42^, but a local and transient effect.

Both InhibinB and AMH are secreted from the GCs of growing follicles. Since most of the growing follicles undergo atresia (follicle apoptosis) and only a few continue to develop toward ovulation, atresia of the vaccine-affected follicles could clarify why the reported menstrual changes are transient. The varying responses to the vaccine among different individuals, might also be influenced by the menstrual cycle phase (follicular or luteal) at the time of vaccination.

The levels of FSH and InhibinB change along the menstrual cycle, but their ratio remains relatively constant, independent of the day of cycle^43^; this ratio is expected to be similar between consecutive menstrual cycles of the same woman. In line with our in-vitro experiments, our in-vivo analysis of the FSH/InhibinB ratio in women before and ~1 month after the 3rd dose of COVID-19 vaccine showed that the post vaccination FSH/InhibinB ratio was changes by 2–3 folds. This change reinforces out hypothesis that vaccination caused an immediate elevation in InhibinB expression, that led to changes in the menstrual cycles’ length and bleeding, as well as to an altered FSH/InhibinB ratio a month later.

As the anti-COVID-19 vaccine is the first commercially available mRNA-based vaccine, and since there is no available vehicle to serve as “control”, we cannot discard the possibility that the changes we characterized in the hpGCs were induced by the vaccine envelope and not specifically by COVID-19 mRNA sequence. Today, when there are more mRNA-based vaccines in the pipeline^44^ this issue is highly relevant.

To summarize, this study reveals a unique, independent mechanism for vaccine related menstrual changes, concomitant with the vaccine-inflicted immune response. Our work suggests that at exposure, the COVID-19 vaccine can affect GCs directly, though not by reducing their viability. Exposure to the end-organ concentration of the vaccine exerted changes in the transcripts of two ovarian-regulatory key factors: a prominent upregulation of *InhibinB* and a downregulation of *AMH*. These changes can strongly affect FSH serum levels in vaccinated women; lead to disrupted follicular growth (i.e., too many follicles growth at the “wrong” time of the cycle) and activity (i.e., estrogen production); and ultimately affect the uterus cyclicity that is clinically displayed by changes in the menstrual bleeding pattern. Serum analysis of vaccinated women who reported menstrual changes, showed a transformed FSH/InhibinB level, supporting our results.

## Methods

The research was conducted in compliance with the Declaration of Helsinki. The Institutional Review Board of Sha’are Zedek Medical Center approved all aspects of the study involving hpGCs (approval number 0240-19-SZMC), and informed consent was obtained from all participants. The Institutional Review Board of Tel Aviv Sourasky Medical Center approved the components of the study related to the menstrual changes following the third dose of the COVID-19 vaccination questionnaire (approval number 0530-21-TLV), for which informed consent was obtained electronically, as well as the serum hormone level measurements (approval number 0086-22-TLV), for which informed consent was obtained from all participants.

### Isolation of human primary granulosa cells (hpGCs)

hpGCs were obtained from Caucasian women, 20–45 years of age with normal BMI, undergoing IVF treatments in Sha’are Zedek Medical Center, Jerusalem between 11/2021 and 12/2022, several months after the administration of the first and second doses of the Pfizer-BioNTech’s COVID-19 vaccine in Israel. Participants’ characteristics are summarized in Supplementary Table 1. hpGCs were isolated from aspirated FF, as previously described^45^. In this methodology of collection, all of the FF is being drawn from the follicle; then, the oocyte is isolated within the cumulus-oocyte complex, most of the remaining cells are mural GCs. FF were centrifuged; the resulting pellets were re-suspended in hemolysis buffer (0.12% Tris, 0.84% NH4Cl) and then washed several times in Dulbecco’s phosphate-buffered saline. The pellets were re-suspended in pools (to ensure an adequate number of cells/well) with fresh culture medium (Dulbecco’s Modified Eagle’s Medium F-12; DMEM-F12; Biological Industries, Beit HaEmek, Israel), supplemented with L-glutamine, penicillin, and streptomycin (2 mM, 10000IU/ml and 100 mg/ml, respectively; Biological Industries) and 10% FBS (Biological Industries). The cells were seeded onto 6-well plates (Thermo Fisher Scientific, Rockford, IL, USA) to allow cell adherence and ensure collection of hpGCs only, and incubated at 37 ^°^C under a controlled atmosphere of 5% CO_2_ in air.

### Stimulation

The following day, hpGCs were washed with fresh culture medium supplemented with the Comirnaty® vaccine for COVID-19 (Pfizer). The pre-clinical trial published in the “Final Report”^20^ by Acutias followed the distribution and accumulation of a LNP vehicle in the body of research-model rats after administration of a single dose, along 48 h^20^, showing that outside the injection site, it was accumulated in 4 out of 28 examined organs of the rats, ovaries being one of them ( ~ 0.1% of injected dose)). In humans, adults are treated with a 30 µg dose/person (equivalent to a ~ 0.006 µg/ml concentration, theoretically distributed in an average 5 L blood volume for the adult women). Thus, we examined two vaccine concentrations (diluted from the original undiluted vile - “stock concentration” of 500 µg/ml): (i) 0.5 µg/ml (“injected dose”), to evaluate cell toxicity. This concentration also mimics ~0.8% of the IM injected dose found in the Plasma 1 h post injection^20^. (ii) 0.5 pg/ml (“end-organ dose”), representing the accumulated concentration in women’s ovaries (~0.1% of the injected dose).

### Calculations of the concentrations used to treat hpGCs

We used Pfizer COMIRNATY purple cup vials. The vial does not state the vaccine concentration, thus we calculated it:

1. Each undiluted vial contains a 225 µg of vaccine in 0.45 ml.
2. The vial is diluted prior to injection with 1.8 ml diluent.
3. The final administrated concentration injected to adults (after dilution) is: 30 µg in 0.3 ml, or 100 µg/ml.
4. Thus, in the diluted vile: 2.25 ml vaccine, at 100 µg/ml, which is equivalent to a total of 225 µg in the vile.

From that we deduced that the undiluted vial contains 225 µg vaccine in 0.45 ml, resulting in a concentration of 500 µg/ml (“stock concentration”).

We used this stock for the treatments as follows:

- “Injected dose”: 1 µl of the vaccine undiluted stock (500 µg/ml) in 1 ml of the cells culture medium results in a concentration of 0.5 µg/ml. This concentration resembles the concentration of the vaccine dose administrated directly to the blood of an adult woman: 30 µg in ~5-liter blood, 0.006 µg/ml.
- “End-organ dose”: 1:1000 dilution of the “injected dose”. This concentration refers to the 0.1% accumulation of the administrated NLP vehicle at the ovaries of female rats as indicated in the “Final report”.

hpGCs were harvested 24 or 48 h later for RNA analysis and the culture medium was collected for protein analysis.

### Cell viability assay

hpGCs were seeded onto a 96-well plate (200 µl/well, in triplicates) in a fresh culture medium and stimulated with the vaccine as elaborated above. Following incubation of 24 or 48 h, hpGCs viability was measured using the MTT assay (M5655; Sigma Aldrich, St. Louis, MO, USA) according to manufacturer instructions^46^.

### Quantitative PCR reaction

RNA was extracted using Trizol reagent (Bio-Tri; Bio-lab Chemicals, Jerusalem, Israel), according to manufacturer’s instructions, and quantified with the Nano-Drop spectrophotometer (ND-1000; Thermo Fisher Scientific, Waltham, MA USA). Total RNA was reverse transcribed using a high-capacity cDNA Reverse Transcription Kit (4368814; Applied Biosystems; Foster City, CA, USA) and used for relative gene expression analysis (20 ng of cDNA per reaction were used as an amplification template). *HPRT1* served as an endogenous normalizing control.

Changes in the mRNA expression levels were detected and analyzed by qPCR, by the StepOnePlus Real-Time PCR System (Applied Biosystems, Thermo Fisher Scientific), using SYBR green reagent (Power SYBR Green PCR Master Mix; Applied Biosystems) and SYBR primers (Table 1) or TaqMan and TaqMan primers (Table 2). Relative expression was calculated using the comparative _Δ_Ct.

**Table 1:** qPCR SYBR primers list

Primer name	Species	Forward (5′to3′)	Reverse (5′to3′)
FSHR	human	GGTGCATTTTCAGGATTTGG	CTGCCTCTATCACCTCCAAGA
HPRT	human	TGACACTGGCAAAACAATGCA	GGTCCTTTTCACCAGCAAGCT
AMH	human	GCATGTTGACACATCAGGC	GAGTGGCCTTCTCAAAGAGC
Aromatase	human	GACTCTAAATTGCCCCCTCTG	CAGAGATCCAGACTCGCATG

**Table 2:** qPCR TaqMan probes list

Primer name	Species	TaqMan^®^ Gene Expression Assay ID
IL-8	human	Hs00174103_m1
InhibinA	human	Hs01081598_m1
InhibinB	human	Hs00173582_m1

### ELISA

Culture medium, collected at the end of the hpGCs’ incubation period, was analyzed by ELISA tests to evaluate InhibinA (InhibinA ELISA; AL-123-I; AnshLabs; Webster, TX; USA) and InhibinB (Ultra-Sensitive InhibinB ELISA; AL-195-I; AnshLabs) protein levels, following manufactures instruction.

### Women’s questionnaire and hormonal analysis

Two hundred forty-eight premenopausal women attending Tel Aviv Sourasky Medical Center for routine physical examinations were asked to complete an electronic questionnaire, inquiring about changes in the menstrual cycle ~4 months after administration of the 3rd dose of the COVID-19 vaccine. Two hundred thirty-six women responded to our request, 124 of which were included in the final analysis (Fig. 4).

### FSH/InhibinB protein level ratio

Hormone levels were measured in the blood of five women (*n* = 5) before and ~1 month after administration of the third dose of the COVID-19 vaccine, that reported changes to their menstrual patterns following the administration of the third dose. We used specific ELISA kits: InhibinB—as in “ELISA for hpGCs”; FSH—Immulite 2000 Xpi (Simense, Berlin, Germany). We compared the FSH/InhibinB ratio, which is relatively constant throughout the menstrual cycle, for each woman in the above mentioned timepoints.

### Statistical analysis

All statistical analyses were performed using the GraphPad Prism 9.0.0. software (GraphPad Software, Inc., San Diego, CA, USA). All presented data is expressed as standard error of the mean. Normality of data distribution was evaluated by Kolmogorov-Smirnov test. To specify significance between experimental groups, we performed a Kruskal–Wallis’s test, followed by FDR correction for multiple comparisons.

## Reporting summary

Further information on research design is available in the Nature Research Reporting Summary linked to this article.

## Supplementary information

Supplementary Information

## Data Availability

All data supporting the findings of this study are included in the main manuscript or the supplementary material. Correspondence and further inquiries for materials can be addressed to D.G. (grisaro@tauex.tau.ac.il).
