# Author Correction: The direct effect of SARS-CoV-2 virus vaccination on human ovarian granulosa cells explains menstrual irregularities

**DOI:** 10.1038/s41541-024-00956-3

**Published:** 2024-09-18

**Authors:** Hadas Bar-Joseph, Yael Raz, Anat Eldar-Boock, Nadav Michaan, Yoel Angel, Esther Saiag, Luba Nemerovsky, Ido Ben-Ami, Ruth Shalgi, Dan Grisaru

**Affiliations:** 1 TMCR Unit, The Veterinary Service Center, Faculty of Medicine, Tel Aviv University, Tel Aviv, 69978 Israel; 2 Department of Gynecologic Oncology, Lis Maternity Hospital, Tel Aviv Sourasky Medical Center, Tel Aviv 6423906, Israel. Affiliated to the Faculty of Medical & Health Sciences, Tel Aviv University, Tel Aviv, Israel; 3 Tel Aviv Sourasky Medical Center, Tel Aviv, Israel, The Faculty of Medical & Health Sciences and the Coller School of Management, Tel Aviv University, Tel Aviv, Israel; 4 Tel Aviv Medical Center, Tel Aviv, Israel; 5 Department of Cell and Developmental Biology, The Faculty of Medical & Health Sciences, Tel Aviv University, Tel-Aviv, Israel; 6 Department of Obstetrics and Gynecology, IVF and Infertility Unit, Sha’are Zedek Medical Center, The Hebrew University Medical School of Jerusalem, Jerusalem, 9103102 Israel

**Keywords:** RNA vaccines, Translational research

Correction to: *npj Vaccines* 10.1038/s41541-024-00911-2, published online 26 June 2024

In the original version of this Article, the vaccine concentrations used in the in vitro experiments were written mistakenly with a 10-fold error. The actual concentration used was 0.5 μg/ml resulting in a 0.5 pg/ml end-organ dose (Discussion). In Methods section Stimulation “of injected dose” was missing after “~0.1%”, and the final concentration of vaccine in blood should have been 0.006 μg/ml. A clarification is added that “This concentration also mimics ~0.8% of the IM injected dose found in the Plasma 1 h post injection.” Throughout the rest of the Methods the stock concentration should have been 500 μg/ml and the injected dose 0.5 μg/ml.

The Article has now been corrected with these changes. This correction does not change the scientific conclusions of the article.

