# Supplementary material for: The direct effect of SARS-CoV-2 virus vaccination on human ovarian granulosa cells explains menstrual irregularities
Source: NPJ Vaccines. 2024 Jun 26;9:117. doi: 10.1038/s41541-024-00911-2 (PMC11208497; doi:10.1038/s41541-024-00911-2)
Supplement: Supplementary file 1 — Supplementary Information [file 41541_2024_911_MOESM1_ESM.pdf]

Supplementary Table 1 – characteristics of hpGCs donors

	Age	IVF cycle number	IVF Protocol	Stimulation	Trigger	FF volume
**Ex1**	37	(-)	Data could not be retrieved	(-)	hCG+GnRH	45
	39	2	Antagonist	r-hFSH and r-hLH	hCG	20
	43	2	Antagonist	r-hFSH and r-hLH	hCG+GnRH	35
	31	(-)	Data could not be retrieved	(-)	hCG+GnRH	65
**Ex2**	24	1	Antagonist	r-hFSH and r-hLH	GnRH	140
	27	4	Antagonist	hFSH and hLH	hCG	45
	37	1	Antagonist	r-hFSH	hCG	50
	26	2	Antagonist	hFSH and hLH	hCG	65
**Ex3**	28	3	Antagonist	r-hFSH	hCG	90
	38	1	Antagonist	r-hFSH and r-hLH	GnRH	90
**Ex4**	44	13	Antagonist	r-hFSH and r-hLH	hCG+GnRH	20
	39	3	Antagonist	hFSH and hLH	hCG	30
	40	3	Antagonist	hFSH and hLH	hCG	50
	24	1	Antagonist	r-hFSH	GnRH	100
**Ex5**	24	1	Antagonist	r-hFSH	hCG	100
	38	1	Agonist-Antagonist	hFSH and hLH	hCG	40
	42	1	Antagonist	hFSH and hLH	hCG	50
**Ex6**	34	2	Agonist-Antagonist	r-hFSH and r-hLH	hCG+GnRH	10
	35	3	Antagonist	hFSH and hLH	hCG+GnRH	10
	26	5	Antagonist	r-hFSH and r-hLH	hCG+GnRH	10
	35	1	Antagonist	r-hFSH and r-hLH	hCG+GnRH	50
	41	8	Antagonist	r-hFSH and r-hLH	hCG+GnRH	10
	39	8	Antagonist	hFSH and hLH	hCG	60
	27	3	Antagonist	hFSH and hLH	hCG+GnRH	20
	39	1	Antagonist	r-hFSH and r-hLH	hCG	10

FF -Follicular fluid volume .

r-hFSH - Recombinant human follicular stimulating hormone. r-hLH - Recombinant human luteizing hormone

hFSH - Human follicular stimulating hormone. hLH - Human luteizing hormone
